# Dermatitis herpetiformis successfully treated with dupilumab

**DOI:** 10.1016/j.jdcr.2025.03.037

**Published:** 2025-05-08

**Authors:** Sara Al-Khawaga, Ashraf I. Ahmed, Fatima Al-Khawaja, Dana Jassim AlShibani, Fatima E. Al-Kubaisi, Samir Al Hyassat, Abdelkareem Alhyari, Joerg Buddenkotte, Martin Steinhoff

**Affiliations:** a Department of Dermatology and Venereology, Hamad Medical Corporation, Doha, Qatar; b Translational Research Institute, Academic Health System, Hamad Medical Corporation, Doha, Qatar; c Dermatology Institute, Academic Health System, Hamad Medical Corporation, Doha, Qatar; d Weill Cornell Medicine-Qatar, Doha, Qatar; e College of Health and Life Sciences, Hamad Bin Khalifa University-Qatar; f Qatar University, College of Medicine, Doha, Qatar; g Anatomic Pathology Division, Department of Laboratory Medicine and Pathology, Hamad Medical Corporation, Doha, Qatar; h Department of Dermatology, Weill Cornell Medicine, New York

**Keywords:** celiac disease, dermatitis herpetiformis, dupilumab, glycogen storage disease

## Introduction

Dermatitis herpetiformis (DH) is an autoimmune blistering disease characterized by pruritic papulovesicular lesions found mostly on extensor surfaces. The cutaneous manifestation is caused by an immunologic response to dietary gluten, leading to the formation of immunoglobulin A antibodies against tissue transglutaminase (TG) and epidermal TG 3 that ultimately deposit in the papillary dermis.^1^ Therapeutic approaches combine a gluten-free diet along with dapsone to control the debilitating nature of the cutaneous and intestinal manifestations of DH. Limited data exists regarding second-line treatments of DH, which makes it challenging to treat those patients with contraindications to dapsone.^2^ Dapsone is metabolized by the liver via acetylation and hydroxylation, producing oxidative metabolites that can exacerbate hemolysis and methemoglobinemia in high-risk patients; thus, it is preferably avoided in patients with glycogen storage disease (GSD), due to their impaired hepatic metabolism and increased risk of oxidative stress.^3^ This is a novel case report demonstrating the successful treatment of DH with dupilumab in a 21-year-old female. Due to an underlying GSD, dapsone was contraindicated, and she was effectively managed with dupilumab 300 mg every 2 weeks.

## Case report

A 21-year-old Middle Eastern female with GSD (type 6 - PYGL mutation) and allergic rhinitis, presented to the general dermatology clinic after a 1-year history of recurrent, painful, itchy vesicular skin rashes. The patient reported rashes that appeared on her trunk, extremities, and gluteal area, lasted for 1 to 2 weeks, and resolved with minimal hyperpigmentation. The current rash was associated with severe itching, ranging from an itch score of 2 to 8/10. The patient noticed no specific triggers and denied any history of wheal-like lesions, shortness of breath, or other angioedema-like symptoms. She also denied any malar rash, mucosal lesions, or nail changes. Nikolsky’s sign for vesicles was negative. During that year, she tried multiple topical treatments, including steroid creams (Clobetasol cream; super potent topical corticosteroid), without significant improvement.

**Fig 1:**
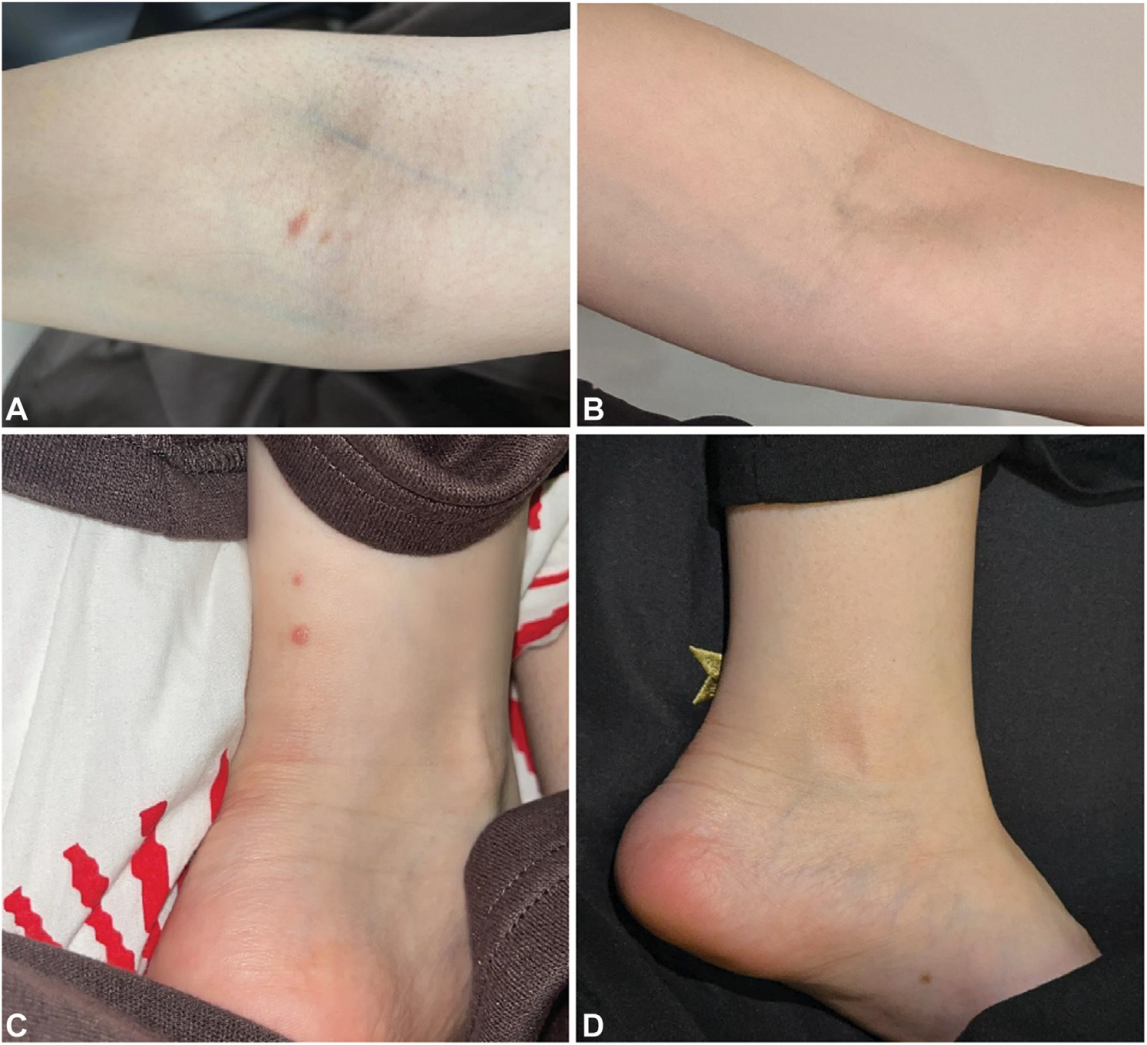
Cutaneous presentation of symmetrically erupted vesicles and papules located on (**A**) arm and (**C**) above the he left medial malleolus, respectively and clinical improvement by dupilumab treatment (**B**, **D**). With kind permission from the patient.

**Fig 2:**
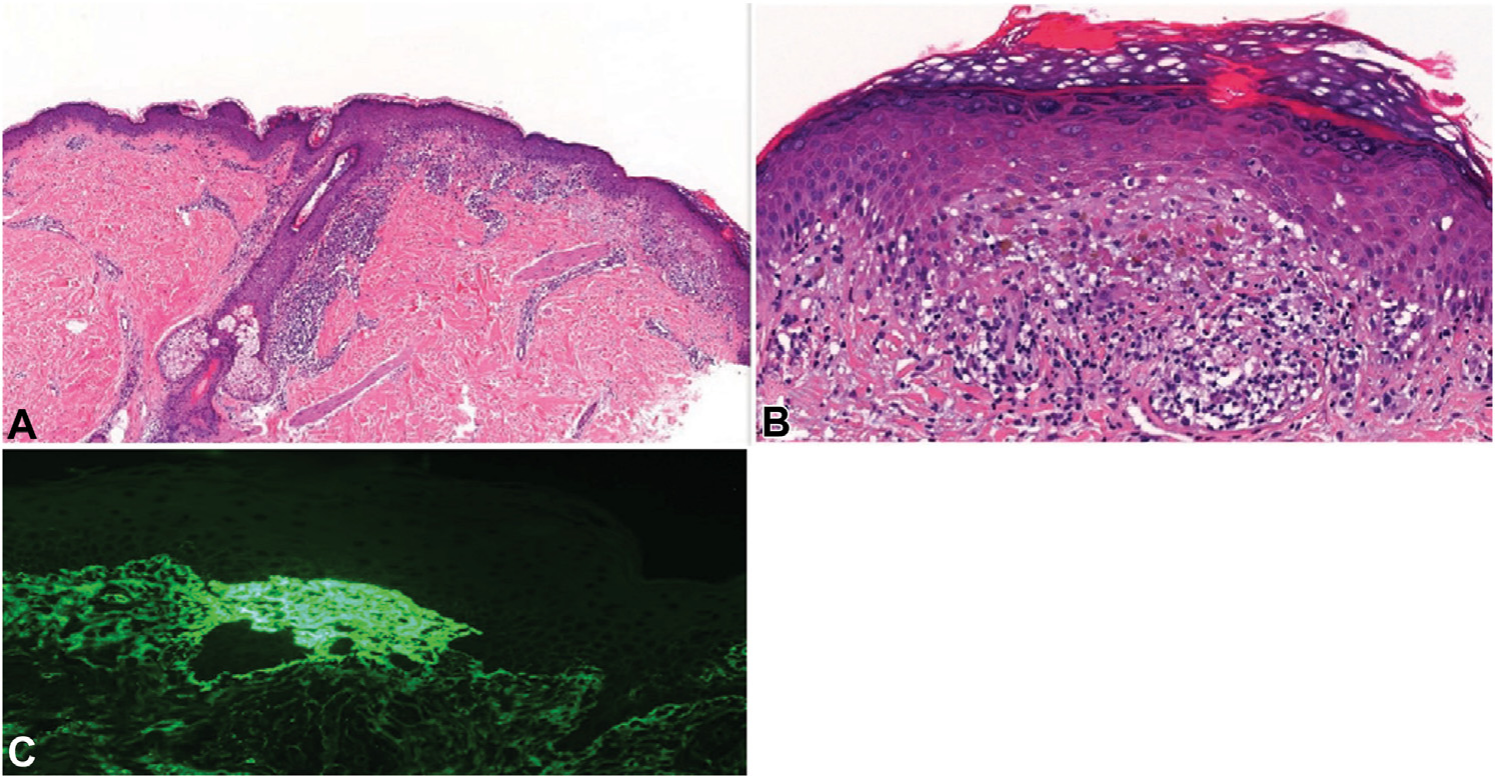
Dermato-histopathological examination of the papule revealed (**A**) upper dermal inflammation (H&E, 2× magnification), (**B**) mild spongiosis with prominent basal vesicular degeneration accompanied with prominent lymphocytic infiltration and focal pigment incontinence inflammation (H&E, 20× magnification), and (**C**) granular immunoglobulin A (IgA) immunofluorescence staining along the dermoepidermal junction and dermal papillae.

Physical examination showed symmetrically erupted papules and vesicles on the extremities (Fig 1, *A* and *C*) and the gluteal region. Results of routine blood work were unremarkable and tests for infections (hepatitis B and C, HIV, and tuberculosis) and autoimmune markers (ANA and connective tissue disease screening), including celiac disease serology (anti-TG antibody) were negative. Upper endoscopy showed unremarkable duodenal mucosa. Microscopic examination of a punch biopsy from lesional skin revealed basketweave orthokeratosis, and moderate to severe spongiosis along with prominent basal vesicular degeneration. Noteworthy findings in the superficial dermis included prominent lymphocytic inflammation and focal pigment incontinence (Fig 2, *A* and *B*). Ancillary studies using immunofluorescence revealed granular and fibrillar basement membrane staining in dermal papillae for immunoglobulin A, immunoglobulin M, C3, C1q, and fibrinogen. However, immunofluorescence studies for immunoglobulin G showed negative results (Fig 2, *C*). Genotyping was negative for HLA DQ2-DQ8. Given the absence of clinical features, negative ANA, and lack of characteristic findings on histopathology and direct immunofluorescence, lupus and lichen planus pemphigoides, among other differential diagnoses, were ruled out in favor of a diagnosis of DH.

Based on clinical and histopathologic findings, the diagnosis of DH was confirmed. Dapsone was excluded as a treatment option due to the patients’ glycogen storage disorder. Tetracycline use was also avoided due to the patient's glycogen storage disease and the associated risk of hepatotoxicity. Therefore, she was initiated instead on subcutaneous injection of dupilumab (300 mg) every 2 weeks with an initial dose of 600 mg. 10 weeks after receiving her first dose, she presented to the clinic with an improved 2/10 itch score and resolved previous lesions but had 2 new lesions on her lower legs. On her third visit (week 19), she presented with zero active lesions and a 0/10 itch score (Fig 1, *B* and *D*). She remained stable and controlled for more than 10 months. After that period, a trial of a 3-week interval of dupilumab instead of a 2-week interval was attempted. However, the patient developed new lesions, and she was placed back on the 2-week interval of dupilumab injection. The patient remained well-controlled with this regimen for 2 years of monitoring with no active lesions or new complaints.

## Discussion

DH is an autoimmune blistering disorder that is effectively managed by dapsone. However, there is no standardized evidence-based protocol in cases where conventional therapy is contraindicated or poorly tolerated (Table I) posing a significant challenge in the management of dapsone-intolerant patients. In the present study, we report a successful off-label use of dupilumab to treat DH. Dupilumab is a monoclonal antibody that targets the IL-4 receptor alpha chain (IL-4Rα), common to both IL-4R complexes.^8^ Our DH patient presented with a contraindication to dapsone due to an underlying GSD (type 6 - PYGL mutation) which necessitated a lifetime therapeutic alternative, in contrast to previously reported cases,^6^ avoiding the risks of dapsone-induced side effects (Table I).^4^^,^^5^^,^^7^ Several treatments including tofacitinib (Janus kinase inhibitor),^4^ rituximab,^5^ and the combination of tetracycline and niacinamide,^6^ or with heparin as triple therapy,^7^ have been used in the management of DH before. However, recent findings of Makino et al (2017) found that patients with DH had elevated serum levels of IL-4, IL-5, and IL-13 compared to healthy subjects.^9^ We thus decided to initiate a more targeted therapy with anti-IL4R dupilumab in our patient. Although the underlying mechanism of DH is till elusive our observation of the clinical benefit of dupilumab may be attributed to a role of Th2-asociated cytokines IL-4 and IL-13 in the pathogenesis of DH. This hypothesis is further corroborated by a finding of Caproni et al reporting elevated RNA expression of the genes encoding for IL-4 and IL-5 in 5 out of 7 investigated patients with DH.^10^ Histopathological examination in addition demonstrated a marked expression of IL-4 in the upper dermis with a predominant perivascular distribution and at sites of dermal-epidermal separation pointing to recruitment of IL-4-positive immune cell in DH.^10^ Our case study together with findings of others support a prominent role of Th2 cytokines in the pathogenesis of DH, adding to the current understanding of an important role of granulocyte macrophage colony-stimulating factor and IL-8 in DH. Dupilumab effectively blocks 2 key Th2 cytokines, IL-4 and IL-13 from binding to their cognate receptor. By inhibiting IL-4/IL-13-mediated signaling, dupilumab can potentially reduce an inflammatory cascade by decreasing the function of T cells in lesional DH skin,^8^ as well as the infiltration of eosinophils and neutrophils into DH lesions. This, in turn, could dampen the inflammatory environment created by Th2 cytokines, which is thought to exacerbate the disease. Given the favorable safety profile of dupilumab, it presents a promising novel therapeutic option for managing disease outcomes in DH patients, particularly when dapsone or other medications are contraindicated.

**Table I:** List of reported cases of dermatitis herpetiformis successfully treated with medications other than dapsone

Medication	Indication	Age	Gender	Celiac disease	Location/description of the lesions	Author
Tofacitinib	Dapsone hypersensitivity syndrome	76	Male	Yes/biopsy proven	Excoriated pink papules and intact small vesicles with clear-yellow fluid on erythematous base scattered on bilateral upper and lower extremities, back, buttock, posterior thigh, and face.	Kahn et al^4^
Rituximab	Worsening anemia after dapsone initiation	80	Male	Not confirmed	Worsening widespread pruritic rash consisting of coalescent erythematous scaling plaques over the arms, legs, back, and buttocks with intermittent scattered vesicles	Albers et al^5^
Tetracycline and niacinamide	Dapsone was not available	42	Male	Not reported	Disseminated erythematous papules on the upper limbs and back as well as vesicles. Nikolsky’s sign for vesicles was negative.	Wang et al^6^
Tetracycline and niacinamide	Dapsone was not available	34	Female	Not reported	Erythematous papules, vesicles, and scabs on the limbs.	Wang et al^6^
Heparin, tetracycline, nicotinamide	Dapsone side effect	42	Male	Not reported	Florid DH with extensive involvement of his chest, shoulders, upper back, legs and feet including numerous bulla	Shah and Ormerod^7^

*DH*, Dermatitis herpetiformis.

## Conflicts of interest

Dr Steinhoff is a consultant for Pfizer, Janssen, Eli-Lilly, Novartis, Abbvie, UCB, Celgene, Galderma, Leo, MenloTx, Sanofi, Regeneron. They received grants by Pfizer, Novartis, Leo, Galderma; and are a speaker for Pfizer, Janssen, Eli-Lilly, Novartis, Abbvie, UCB, Celgene, Galderma, Leo, MenloTx, Sanofi, Regeneron. Buddenkotte and Al-Khawaga were investigators in a Novartis-sponsored clinical study. Drs Ahmed, Al-Khawaja, AlShibani, Al-Kubaisi, Al Hyassat, and Alhyari have no conflicts of interest to declare.
